# Current News

**Published:** 1902-05

**Authors:** 


### Current News.

### AMERICAN MEDICAL ASSOCIATION, SECTION ON STOMATOLOGY.

At the meeting of the Section on Stomatology of the American Medical Association, to be held at Saratoga Springs, N. Y., June 10 to 13, 1902, the following programme will be given:

1. Chairman's Address, Dr. A. H. Peck, Chicago, Ill.
2. "The Embryology of the Dental Pulp," Dr R. R. Andrews, Cambridge, Mass.
3. "The Histology of the Pulp," Dr. Vida A. Latham, Chicago, Ill.
4. "Notes on the Preparation of Teeth for the Microscope," Dr. Martha Anderson, Moline, Ill.
5. "Evolution of the Pulp," Dr. Eugene S. Talbot, Chicago, Ill.
6. "A Comparative Study of the Attachment of Teeth," Dr. Frederick Noyes, Chicago, Ill.
7. "Permanent Benefit resulting from the Correction of Irregularities of the Teeth due to Interstitial Gingivitis," Dr. M. H. Fletcher, Cincinnati, Ohio.
8. "Observations on some Recent Cases of Orthodontia, with Illustrations," Dr. E. A. Bogue, New York City.
9. "General Nervous Manifestations in Relation to the Jaws and Teeth," Dr. G. V. I. Brown, Milwaukee, Wis.
10. "Electric Ozonation upon Neuralgia," Dr. G. Lenox Curtis, New York City.
11. "Diagnosis," Dr. Jonathan Taft, Cincinnati, Ohio.
12. "The Modern Dentist from a Medical Stand-Point," Dr. William Knight, Cincinnati, Ohio.
13. "Chancre of the Lip," Dr. G. T. Carpenter, Chicago, Ill.
14. "Oral Hygiene," Dr. G. F. Eames, Boston, Mass.
15. "The Legal Status of the Term 'Reputable' as applied to Dental Colleges," Dr. Charles Chittenden, Madison, Wis.
16. "Autoinfection of the Mouth," Dr. G. L. Parmele, Hartford, Conn.

17. "Dento-Facial Orthopædia," Dr. W. E. Walker, New Orleans, La.

Dentists desiring to become members of the Section can do so by obtaining credentials from their State or local dental society and presenting them with the sum of five dollars to the Treasurer of the Association. This sum includes the Journal of the Association for one year. All dentists are invited to attend and take part in the discussions.

A. H. Peck,
*Chairman.*
Eugene S. Talbot,
*Secretary.*

---

### MISSOURI STATE DENTAL ASSOCIATION.

The thirty-eighth annual session of the Missouri State Dental Association will convene at Jefferson City, Mo., May 21, 22, and 23, 1902. The literary programme will be held in the Legislative Hall, and the clinics, beginning at ten a.m. the first day, will be held at the State Penitentiary, where an abundance of clinical material can be had.

A cordial invitation is extended to all reputable dentists to attend. Railroad and hotel rates have been secured.

The following is a partial list of the programme:

ADDRESSES AND ESSAYS.

1. Burton Lee Thorpe, St. Louis, President's Annual Address.
2. Wm. Everett Griswold, New York, "The Griswold System of Removable Bridge-Work."
3. Frederick Brown Moorhead, Chicago, "Alveolar Abscess: Its Sequel and Surgical Treatment."
4. D. R. Stubblefield, Nashville, Tenn., "Metallurgy."
5. J. D. Patterson, Kansas City, "Etiology of Dental Disease."
6. D. F. Luckey, D.V.S., Missouri State Board of Agriculture, Columbia, "Comparative Anatomy of the Teeth."
7. J. Robert Megraw, Fayette, "Dental Prescriptions."
8. Millard Lewis Lipscomb, A.M., Missouri State University, Columbia, "The Practical Application of Electricity in Surgery and Kindred Subjects."
9. S. C. A. Rubey, Clinton, "Some State Board Questions and the Answers they receive."

10. James W. Hull, Kansas City, "Conservatism in Dentistry."

11. Herman Prinz, St. Louis, "Some of the Newer Dental Remedies."

12. Charles Gilbert Chaddock, M.D., St. Louis, "Neurology."

13. W. W. Flora, Carthage, "Use and Abuse of Crown- and Bridge-Work."

14. Otto J. Fruth, St. Louis, Report of Committee on New Inventions and Appliances.

15. H. S. Vaughn, Kansas City, "Orthodontia."

CLINICS.

[The clinical work covers thirty-eight operations, distributed over pathological and practical cases, but too extended to be admitted in full in our limited space.—Ed.]

GEO. W. TAINTER, Chairman, Jefferson City,
C. D. LUKENS, St. Louis,
J. C. PASQUETH, Mexico,
*Executive Committee.*

---

### PENNSYLVANIA BOARD OF DENTAL EXAMINERS.

THE Board of Dental Examiners of Pennsylvania will conduct examinations in Philadelphia, June 24 to 27, 1902.

For papers and further information, address Hon. James W. Latta, Secretary Dental Council, Harrisburg, Pa.

G. W. KLUMP,
*Secretary.*

WILLIAMSPORT, April 5.

---

### MISSISSIPPI BOARD OF DENTAL EXAMINERS.

THE Mississippi Board of Dental Examiners will hold its annual meeting in the city of Jackson, on Tuesday, May 27, 1902.

J. P. BROADSTREET,
*President.*
W. R. WRIGHT,
*Secretary.*

